# The Perception of Trustworthiness and Emotional Identification in Women Experiencing Intimate Partner Violence: A Behavioral Pilot Study

**DOI:** 10.3390/brainsci15050429

**Published:** 2025-04-23

**Authors:** Valentina Silvestri, Silvia Gobbo, Erica Pugliese, Francesco Mancini, Federica Visco-Comandini

**Affiliations:** 1 Department of Psychology, University of Milan-Bicocca, 20126 Milano, Italy; v.silvestri11@campus.unimib.it (V.S.); silvia.gobbo@unimib.it (S.G.); 2 Department of Clinical Psychology, University of Amsterdam, Nieuwe Achtergracht 129-B, 1018 WS Amsterdam, The Netherlands; e.pugliese@uva.nl; 3 Associazione di Psicologia Cognitiva APC e Scuola di Psicoterapia Cognitiva SPC, 00185 Roma, Italy; f.mancini@unimarconi.it; 4 Department of Human Science, Marconi University, 00193 Rome, Italy

**Keywords:** trustworthiness, face processing, emotion, violence, intimate partner violence, pathological affective dependence

## Abstract

Background: Research indicates that traumatic events, such as interpersonal violence, can significantly affect how individuals perceive facial characteristics and assess trust. This study aims to explore trustworthiness and emotional perception in women experiencing intimate partner violence (IPV). Methods: Twenty-four women who have experienced IPV and twenty-four control participants completed an online task. They rated the trustworthiness of male and female faces, chose the more trustworthy face in a pairwise task, and identified emotions displayed by faces. Results: The results revealed that survivors of IPV showed lower accuracy in trustworthiness judgments, particularly for male faces, and in recognizing fear in male faces, compared to the control group. Conclusions: These preliminary findings, constrained by the limited sample size, are discussed in the context of the Pathological Affective Dependence theory and a perceptual model of social face perception, shedding light on the complex interplay between trauma, social perception, and emotional processing.

## 1. Introduction

Humans’ understanding of the world is highly influenced by the rapid and automatic evaluation we make of others based on facial appearance. Since the earliest hours of life [1,2], humans are sensitive to the so-called changeable aspects of faces, such as eye gaze or expressions, which allow us to infer other people’s emotional status, mental state, or intention, which is particularly important for social communication [3,4,5].

By observing the face, it is possible to spontaneously, automatically, and systematically form “first impressions” to adapt one’s behavior during social interactions [5]. One of the first impressions that is generated is facial trustworthiness, that is, whether others are likely to approach in a friendly or hostile manner, and whether we can trust them or not [6,7]. This impression allows humans to deduce people’s intentions and orient their behavior toward either approach or avoidance [8]. Several studies have shown that trustworthiness judgments are made as fast as 100 milliseconds (ms) after exposure to novel faces and correlated with those made without time constraints [9]. Moreover, Todorov and colleagues [10] showed that trustworthiness discrimination was also possible for faces presented at a subliminal level, as fast as 33 ms.

Although the neural and functional mechanisms underlying emotion identification and trustworthiness judgments partially overlap, they represent different concepts. According to the Emotional Overgeneralization Hypothesis [11], there is a tendency to overgeneralize approaching or avoidant behaviors towards others whose faces recall a happy or angry facial expression, respectively [12]. Therefore, even though there certainly is an overlap between the action units necessary for trustworthiness and emotion perception, and between their neural correlates, trustworthiness judgments can also be performed with emotionally neutral faces.

Taken together, this evidence reveals that sensitivity to facial cues of trustworthiness and emotion arises from both innate and experiential factors, reflecting an evolutionary adaptation that helps distinguish between friendly and hostile intentions. This ability is further refined through social experiences over time [7,9,13].

Specifically, the personal experience in the social context in the first years of life modulates emotional perception, as in the case of the attentional bias for threatening stimuli of maltreated children [14]. Likewise, in terms of emotional discrimination, war veterans also appear to have difficulties, primarily related to the hyper-arousal symptoms of post-traumatic stress disorder (PTSD). Indeed, individuals with PTSD show a hyper-vigilance to threatening stimuli, which leads to preferential attention and increased reactivity to this class of stimuli in order to identify them and adapt their response to the aversive environment [15].

A category particularly relevant to this study is that of survivors of Intimate Partner Violence (IPV), which has been extensively regarded as a global problem and a significant issue impacting human rights and public health [16]. According to the United Nations [17], violence against women can include any act of gender-based violence that causes or is likely to cause physical, sexual, or mental harm or suffering to women, including threats of such acts, coercion, or arbitrary deprivation of liberty, whether in public or private life. Recent data from the World Health Organization and the Centers for Disease Control and Prevention reveal that approximately 1 in 3 women globally have experienced either physical or sexual violence in their lifetime [18]. IPV is insidious and dangerous, given that the harm is coming from someone who should love and protect. Recently, IPV has been associated with Pathological Affective Dependence (PAD), a psychological condition that explains the pathological connection between the abusive partner and the survivor [19,20]. According to the PAD theory, women directly experiencing IPV face a conflict between the need to separate from their abusive partner and the need to remain with or return to the abusive partner. Survivors of IPV tend to go back and forth between these two main relational needs, without any solution. The theory of Pathological Affective Dependence (PAD) is based on the interaction between distal and proximal factors that influence behavior in abusive relationships. Distal factors are the underlying causes that give rise to PAD, such as unmet needs for love, dignity, and security, often frustrated in relationships with parents or partners. These unmet needs give rise to proximal factors, which are psychological barriers that compel individuals to stay in abusive relationships and are the main characteristics of PAD. These barriers include internal conflict, the inability to separate from an abusive partner, and partner abuse [21]. Also, although some survivors leave abusive relationships, others are unable to do so. Some authors explain the former condition as a consequence of factors such as external support, fear of the harm that the abuser can cause, and the desire to protect their children [22,23]. Other authors believe that the reason why survivors stay can be explained by the difficulty in disengaging from such relationships and the possibility that they may underestimate the potential danger associated with persisting in the relationship with their abusers, as a consequence of a reduced ability to perceive pain after being repeatedly battered [24]. There are other factors impacting the possibility of disengaging from violent relationships, such as economic abuse, impaired access and use of financial resources [25], perception of self-efficacy [26], responsibility for children [27], presence of secure and protective services and legal mechanisms [28], preexisting inequities, particularly for families from marginalized communities, and the recent COVID-19 spread [29]. These barriers to ending a violent relationship should not be underestimated. Instead, they should be understood within a broader context that encompasses both individual and external factors. Psychological conditions such as PAD, PTSD, Complex PTSD (cPTSD), and Borderline Personality Disorder (BPD) significantly influence the decision to remain in or leave an abusive relationship [30,31]. These psychological conditions may also be associated with other individual factors, such as an overestimation of trustworthiness from others. These types of relationships have negative consequences on mental and physical health [32,33,34,35,36,37,38] and a higher risk of difficulty with emotional recognition [39]. Yet, recognizing dangerous situations is a fundamental skill for responding effectively in high-risk situations and identifying signals of imminent risk of victimization [40,41]. Given the impact that traumatic relationships may have on social cognition, the present study aims to deepen our understanding of emotional and facial trustworthiness perception in survivors of IPV. In particular, we investigate whether and how prolonged exposure to violence may influence automatic social perception processes, which are fundamental for evaluating others’ intentions and ensuring personal safety in social interactions. Indeed, different studies suggest that survivors may not be able to differentiate between threatening and typical behavior commonly associated with intimate relationships [40,41]. Moreover, where emotion recognition is concerned, Depierro and colleagues [42] revealed that survivors of IPV exhibited a bias toward IPV trauma-related words (e.g., violence, attack, or assault), compared to neutral (e.g., stamp, circle, or format) or positive words (e.g., goodness, peaceful, or hopeful) [43,44,45]. Survivors of IPV manifested a facilitation in IPV-related word recognition compared to controls and reported increased symptoms of post-traumatic stress disorder (PTSD). Also, Clauss and Clements [46] showed that survivors of IPV demonstrated less emotional recognition to only happy faces. They also revealed that survivors of IPV showed a threat bias, with survivors of IPV attending heavily to threatening stimuli and having difficulty in disengaging from threatening images. This evidence indicates the importance of recognizing fearful faces, as women who directly experienced IPV showed no difference in accuracy compared to the control group. This absence of difference leads to the consideration that threat bias (i.e., the difficulty in attentional disengagement from threatening images) cannot be explained by recognition ability. The protracted attention to threatening faces and the difficulty in disengaging from these stimuli were explained by the authors as a common behavioral pattern in abusive relationships.

Where trustworthiness is concerned, the evidence has shown differences in this domain in IPV victims. Particularly, only women directly experienced with IPV underestimated male faces on the dominance dimension and overestimated them on the trustworthiness dimension, thus judging male faces as more trustworthy. These results can be understood as a consequence of the habituation process: women who directly experienced violence for an extended period from partners and attachment figures with dominant personalities tend to underestimate dominance and overestimate trustworthiness in faces. These findings may also reflect the activation of a protective mechanism to survive violence by adopting submissive behaviors [47]. Furthermore, these behaviors may be related to traumatic bonding [48,49], where the survivors’ emotional attachment to the abuser becomes a powerful force that complicates their ability to leave the relationship. This need to remain with or return to an abusive partner aligns with the concept of Pathological Affective Dependence (PAD).

This evidence highlights the importance of accurately recognizing both risky situations and emotions. The ability to identify emotional signals has the potential to contribute to the prevention of engaging with an abusive partner and remaining in a violent relationship. This is achieved when individuals can accurately identify threatening or violent partners and ensure their safety by leaving a dangerous situation before the violence evolves into homicide. Within this view, it is essential not only to identify emotional faces rapidly but also faces expressing traits associated with threat or trustworthiness. These serve as signals of potential danger or threat, prompting the need to maintain a safe distance. Thus, given that the influence of life experience on perception remains unexplored in the field of cognitive science, it is pivotal to deeply investigate trustworthiness and emotional perception in women who have experienced IPV by assessing the facial gender as a potential factor impacting the perception of a face as threatening.

Thus, the present study aims to replicate and extend the evidence on trustworthiness and emotional perception in women who have experienced IPV, compared with women who do not explicitly report having experienced violence. Furthermore, since the perception of trustworthiness overlaps with emotional recognition, and given the evidence on different emotional recognition abilities in trauma victims compared to non-victims, the present study also aims to investigate emotional recognition in women who have experienced IPV, compared to those who do not report being survivors of IPV. Compared to prior investigations focusing on the perception of trustworthiness [47] and emotions in IPV [42,46], the current study endeavored to delve into how facial gender influences facial perception. We hypothesized that, given that violence experienced by women is predominantly perpetrated by individuals identifying as male (i.e., the partner; [33]), a specific impairment in face processing only concerns male faces but not female ones. Lastly, in addition to examining the perception of trustworthiness and emotional recognition, we aimed to account for psychological factors that may influence facial perception in women with a history of IPV. Indeed, to control for individual differences that could influence the perception of trustworthiness and emotional recognition, we included psychological measures, including alexithymia, post-traumatic stress disorder symptoms, and depressive symptoms. Specifically, we included scales to assess alexithymia, PTSD symptoms, and depressive symptoms, as these factors are known to affect emotional processing and could provide a deeper understanding of the perceptual biases observed in survivors of IPV.

## 2. Materials and Methods

### 2.1. Transparency

Here, we report how we determined our sample size, all data exclusions, all manipulations, and all measures in this study, following JARS [50]. The data were analyzed using Jamovi software v2.3.21. (https://www.jamovi.org). All data have been made publicly available at the OSF and can be accessed at (https://osf.io/t69he/?view_only=196e15e2fdd647b681a64233d03ebe0c, accessed on 1 January 2022). This study was not preregistered.

### 2.2. Participants

The participants were 48 women recruited online and through word of mouth. A total of 24 women (Mage = 45, SDage = 8.45) comprised the sample who experienced IPV and were recruited through anti-violence centers in the SanFra network, to which they were afferent through the collaboration of Dr. Anna Moschettini, Marika Massara, Fabiana Perosce, and Michela Garzia, and represented the experimental group. All the participants were housed within institutional shelters for women who have been victims of violence, and therefore, all had been exposed to interpersonal violence of a physical, sexual, or psychological nature. A total of 24 women (Mage = 35, SDage = 14.01) constituted the sample of women who reported not having experienced violence in their lifetime (no IPV). The participants in the control group were screened to ensure they had no history of trauma or interpersonal violence.

The sample was determined using a priori power analysis, no sequential testing was performed, and data were not analyzed until the data collection was completed. Indeed, a power analysis for a repeated measure ANOVA with two within factors (Trustworthiness and Gender) and one between factor (Group of women) revealed that 46 participants would be needed to have a 90% chance of observing a significant effect with an alpha level of 0.05 and a medium effect size.

All the participants provided written informed consent before participating in this study. This study was performed in accordance with the WMA Declaration of Helsinki (BMJ 1991; 302: 1194) for research involving human subjects, and the protocol was approved by the Ethics Committee (Protocol N. 5/2024) at the School of Cognitive Psychotherapy of Rome (Italy).

### 2.3. Stimuli and Procedure

The participants provided their ratings through an online questionnaire, divided into different sections corresponding to different tasks and delivered through the platform Qualtrics (Qualtrics, Provo, UT, USA, https://www.qualtrics.com).

They all ran the experiment on a desktop located in the administration office of the reception center where all the participants were living (in the IPV group) or on the desktop of their personal computer (no-IPV group). They were instructed to sit approximately 30 cm from the monitor (20 × 26.5 cm and 13 inches in size, with a resolution of at least 1280 × 800 pixels).

#### 2.3.1. Pairwise Preference Task

The stimuli consisted of 12 images of faces belonging to 6 female identities of Caucasian origin (3 judged as low trustworthy, LT, and 3 as high trustworthy, HT) and 6 male identities of Caucasian origin (3 judged as low trustworthy, LT, and 3 as high trustworthy, HT). The faces were selected from the Karolinska Directed Emotional Faces (KDEFs) Database [51]. In this task, the participants saw on each trial two faces randomly selected from the same trustworthiness continuum simultaneously appear on the screen, and they were asked to select the face they trusted more by selecting it with the mouse, as in [52,53]. These 2 faces were randomly selected from a group of 6 faces per gender, each with a different and growing level of trustworthiness. Trustworthiness levels were decided based on the trustworthiness rating expressed in the validation of the Chicago Face Database images [54], where participants were asked to rate the trustworthiness level of each face on a Likert scale ranging from 1 (not trustworthy at all) to 7 (extremely trustworthy). We selected six faces for the female and male groups, respectively, thus creating a continuum ranging from “untrustworthy” to “trustworthy”.

**Figure 1:**
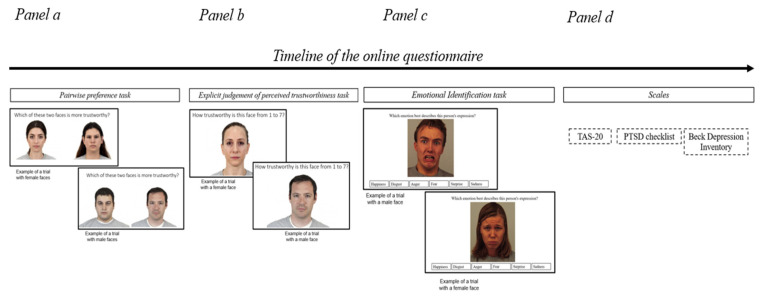
Example of the procedure in the online questionnaire, with each task presented to the participants. (**Panel a**) represents the pairwise preference task. (**Panel b**) represents the explicit judgment of the perceived trustworthiness task. (**Panel c**) represents the emotional identification task. (**Panel d**) represents the three scales proposed at the end of the questionnaire.

The participants completed 16 trials per gender, corresponding to all possible pairwise combinations of the six faces of the continuum (32 trials in total). Each face of the continuum was compared to all other faces of the same continuum for a total of five times; the position of the faces on the screen was randomized across trials, and trials within each block were presented in random order. An example of the experimental task can be seen in Figure 1, Panel a.

#### 2.3.2. Explicit Judgment of Perceived Trustworthiness Task

The stimuli were the same faces as those described in the pairwise preference task. Participants accessed the online questionnaire, during which 12 faces (6 male, 3 untrustworthy and 3 trustworthy, and 6 female faces, 3 untrustworthy and 3 trustworthy) were shown one by one, and were asked to indicate the degree of trustworthiness on a 7-point Likert scale, where 1 indicated very untrustworthy and 7 indicated very trustworthy. Subjects were told to respond by “using their gut”. They started each trial by pressing the mouse, and the stimuli remained on the screen until a response was made. The stimuli were presented in random order. An example of the experimental task can be seen in Figure 1, Panel b.

#### 2.3.3. Emotional Identification Task

Emotional faces were selected from the Karolinska Directed Emotional Faces (KDEFs) Database [51] and were validated in a further validation study.

We ran a further validation study to ensure that the faces we selected better reflected the emotions expressed. We asked 22 adults (6 females, mean age = 34.5, SD = 10.8) to indicate the emotion expressed by the face and its intensity on the basis of a 7-point Likert scale (from 0, no intensity, to 7, extreme intensity). We presented the adult participants with 48 faces, 24 female identities, and 24 male identities, 6 for each of the six basic emotions [55]. Of the presented 48 faces, the 24 more frequently identified as correct and with the greatest intensity were selected for the main task (12 females). The participants provided their ratings through an online questionnaire delivered on Qualtrics (Qualtrics, Provo, UT, USA, https://www.qualtrics.com). A Friedman’s test showed statistically significant differences (*p* < 0.001) in emotional valence judgments among the emotions, specifically between sadness and disgust (*p* < 0.001), between sadness and threat (*p* = 0.006), between disgust and surprise (*p* = 0.002), and between disgust and anger (*p* = 0.007). Similarly, participants were less accurate in identifying fearful faces than all other emotions (all *p* < 0.001). It should be considered that different studies showed that not all emotions are identifiable in the same manner with the same intensity and accuracy [56]. As our aim is, precisely, to investigate possible gender differences in emotion perception with IPV and no-IPV, for the purpose of the present study, the most relevant control relates to the perceived difference for each emotion between male and female faces. We controlled so that ratings did not significantly differ based on the gender of the face. A Wilcoxon test revealed no statistically significant difference in intensity (all *p* > 0.15) and accuracy (all *p* > 0.09) between male and female faces.

Based on these stimuli, the participants (i.e., IPV and no-IPV) were asked, for each face, to identify which of the six basic emotions was displayed (i.e., fear, anger, sadness, happiness, surprise, and disgust, from [51]). We used faces validated by the adult sample described above. An example of the experimental task can be seen in Figure 1, Panel c.

#### 2.3.4. Scales

Finally, the participants were asked to complete the adapted form of the TAS-20 [57,58], the PTSD checklist [59], and the Beck Depression Inventory-Revised (BDI-II, [60]). The Toronto Alexithymia Scale (TAS-20) was administered to explore any problems linked to emotional expressions in the sample. TAS-20 is the main instrument for self-assessment, validated in Italian [61], of the construct of alexithymia, defined as the set of characteristics involving difficulty in identifying and expressing feelings, an absence of imagination, a style of concrete and outward-oriented thinking, and a difficulty in differentiating between emotions and body sensations [62,63]. The respondents rated 20 items on a 5-point Likert scale, with higher scores indicating greater alexithymia. The scale has been validated in Italian and has demonstrated good reliability and validity in both clinical and non-clinical populations. To control for the presence of any PTSD symptoms, we used the PCL, validated in Italian [64]. The checklist consists of 20 items that reflect the DSM-IV criteria for PTSD, with respondents rating how much they have been bothered by each symptom during the past month on a 5-point scale (from 1 = Not at all, to 5 = Extremely). The PCL has been validated in Italian and is commonly used to evaluate the presence and severity of PTSD symptoms. Higher total scores indicate more severe symptoms of PTSD. Finally, to assess the possible presence and severity of depressive symptoms in our sample, we used the BDI-II, validated in Italian [65]. The BDI-II is a 21-item self-report scale used to assess the severity of depressive symptoms. Each item consists of four statements, with respondents choosing the one that best describes their experience during the last two weeks. A representation of the experimental procedure can be found in Figure 1, Panel d.

## 3. Results

### 3.1. Statistical Models

To investigate differences between the sample of women who have experienced IPV and the no-IPV controls, we ran three different exploratory analyses for each task: one for the explicit judgment of perceived trustworthiness, one for the preference judgment of perceived trustworthiness, and one for emotional identification. Thus, the dependent variables in the three analyses for the three different tasks were the mean explicit judgment of perceived trustworthiness assigned to the high-trust and low-trust faces in male and female conditions, the accuracy in the pairwise preference task in identifying the most trustworthy face in the continuum, and accuracy in the emotional identification task. Additionally, in our exploratory analysis, we included in all our statistical models, including TAS-20, PTSD checklist, and BDI scores, as covariates.

A difference in the identification of trustworthy male and female faces in the IPV group was guided by the hypothesis of the whole study, as we planned to explore the possible differences between the male and female faces within each group through an independent sample *t*-test.

All analyses were performed through the software Jamovi (version 2.3.28, retrieved from https://www.jamovi.org) and Jasp (version 0.17.2.1, retrieved from https://jasp-stats.org/).

#### 3.1.1. Accuracy in the Pairwise Preference Task

To explore how the gender of faces affected the likelihood that the participants selected the more trustworthy face in the pair, the participants’ response accuracy was used as a dependent variable in a repeated measures ANOVA with the gender of stimuli (male and female faces) as the within-subject factor and the group (Controls and Survivors) as between-subject factor.

The analysis revealed a significant main effect of the gender of the faces, F_(1,46)_ = 8.102, *p* = 0.007, and η^2^_p_ = 0.150, with the participants being less accurate with male (M = 75; SD = 14.91) compared to female faces (M = 81.53, SD = 12.55). No main effect of the group was observed, F_(1,46)_ = 1.33, *p* = 0.255, and η^2^_p_ = 0.028. No two-way interaction between the gender of the faces and the group was found, F_(1,46)_ = 0.297, *p* = 0.588, and η^2^_p_ = 0.006.

**Figure 2:**
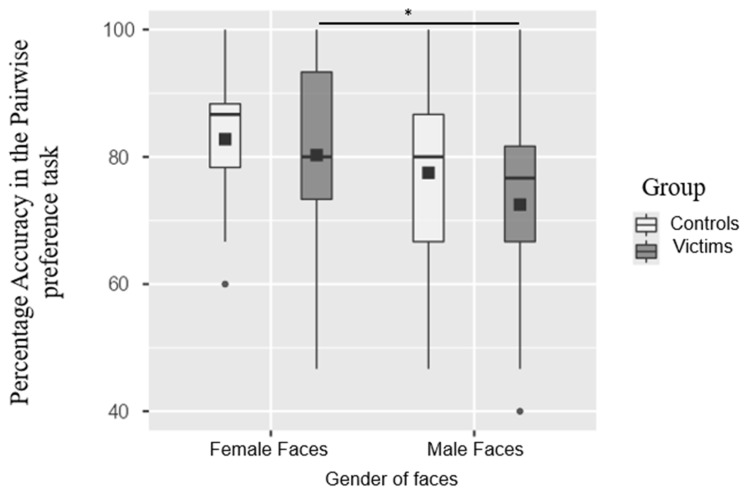
Boxplot representing the mean values in the pairwise preference task. The error bars refer to the standard errors of the group mean, displayed with black squares, The black square represents the mean, the line indicates the median, and the dots are the outliers. * *p* < 0.05. ** *p* < 0.005. *** *p* < 0.001.

A difference in the identification of trustworthy male and female faces in the IPV group was guided by the hypothesis of the whole study, as we planned to explore the possible differences between the male and female faces within each group through a paired sample *t*-test. We found no statistically significant difference between the male (M = 77.5, SD = 14) and female faces (M = 82.8, SD = 10) accuracy in the control group (t(23) = 1.86, *p* = 0.07, d = 0.38). On the contrary, we found a statistically significant difference in the mean accuracy, with better identification of trustworthiness from the female (M = 80.3, SD = 14.8) compared to the male faces (M = 72.5, SD = 15.6) in the IPV group (t(23) = 2.16, *p* = 0.04, d = 0.44), as shown in Figure 2.

#### 3.1.2. Explicit Judgment of Perceived Trustworthiness

**Figure 3:**
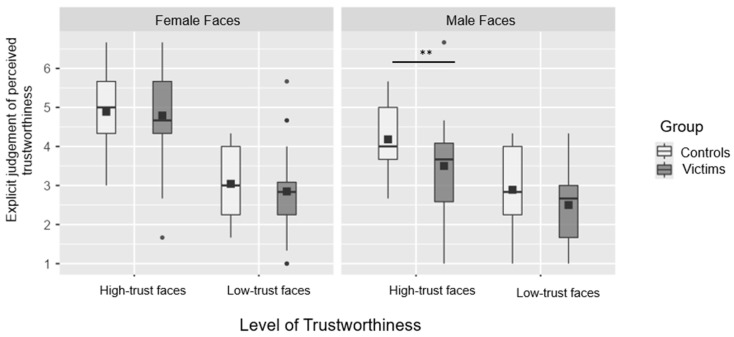
Boxplot representing the mean values in the explicit judgment of the perceived trustworthiness task. The error bars refer to the standard errors of the group mean, displayed with black squares, the line is the median and the black square is the mean and the black dots are the outliers. * *p* < 0.05. ** *p* < 0.005. *** *p* < 0.001.

A repeated measures analysis of variance (ANOVA) was conducted, where the mean of the explicit judgment of perceived trustworthiness was set as the dependent variable, the trust level (high vs. low) and the gender of the seen face (female vs. male) were set as within-subjects factors, while the group (survivors vs. controls) was set as the between-subject factor. A main effect of trust level was found (F_(1,46)_ = 120.86, *p* < 0.001, and η_p_^2^ = 0.72) where the faces with a high trust (M = 4.34; SD = 1.15) were judged as more trustworthy with respect to those with low trust (M = 2.82; SD = 1.03). Moreover, a main effect of gender of the faces was found (F_(1,46)_ = 38.54, *p* < 0.001, and η_p_^2^ = 0.46) where female faces are judged as more trustworthy (M = 3.89; SD = 1.41) with respect to male faces (M = 3.27; SD = 1.17). No main effect of group was observed (F_(1,46)_ = 2.16, *p* = 0.15, and η^2^_p_ = 0.04). In addition, a significant interaction between trust level and the gender of the stimulus was found (F_(1,46)_ = 26.96, *p* < 0.001, and η_p_^2^ = 0.37). All post hoc comparisons resulted as significant (i.e., high-trust male faces vs. low-trust male faces t(46) = 7.34, *p* < 0.001; high-trust female faces vs. low-trust female faces t(46) = 12.18, *p* < 0.001; high-trust male faces vs. high-trust female faces t(46) = −8.07, *p* < 0.001; and low-trust male faces vs. low-trust female faces t(46) = −2.02, *p* = 0.04). It might be that the significant difference between high-trust male faces and high-trust female faces is driven by the control group, even if we did not find a main effect of the group. This might be due to the fact that a larger sample size would be needed and might also drive the non-significant triple interaction between the gender of the stimulus, the trust level, and the group (F_(1,46)_ = 1.81, *p* = 0.18, η^2^_p_ = 0.04). This is why we carried out an analysis to speculate on this. We conducted a simple effect follow-up analysis to further investigate possible differences between the controls and survivors, as this was the main aim of the present study. The results of the simple effects show that the survivors rate high-trustworthy male faces as less trustworthy (M = 3.50, SD = 1.16) than the controls (M = 4.18, SD = 0.81) (F_(1,46)_ = 5.56, *p* = 0.02). This result is displayed in Figure 3. No other statistically significant differences between the survivors and controls were found (all *p* > 0.18).

### 3.2. Emotional Identification Task

#### 3.2.1. Accuracy

To explore how the gender of the faces affected the accuracy in selecting the emotional faces, the accuracy was entered as a dependent variable into a repeated-measure ANOVA. We included the gender of faces (male and female) and emotion (six basic emotions) as within factors, and the group (controls and survivors) as between factors.

The analysis revealed a significant main effect of the gender of the faces, F_(1,46)_ = 6.116, *p* = 0.002, and η^2^_p_ = 0.117, with higher accuracy with the female (M = 89.23; SD = 24.10) rather than the male (M = 85.42, SD = 27.92) faces. We found a main effect of emotion, F_(5,230)_ = 19.852, *p* < 0.001, and η^2^_p_ = 0.301, with fearful faces identified with less accuracy compared to all the other emotions (all *p* < 0.001).

These main effects were both qualified by a significant two-way interaction between these two factors (F_(5,230)_ = 2.365, *p* = 0.041, η^2^_p_ = 0.049). Finally, we found a significant three-way interaction between the gender, the emotion, and the group, F_(5,230)_ = 2.596, *p* = 0.026, and η^2^_p_ = 0.053.

**Figure 4:**
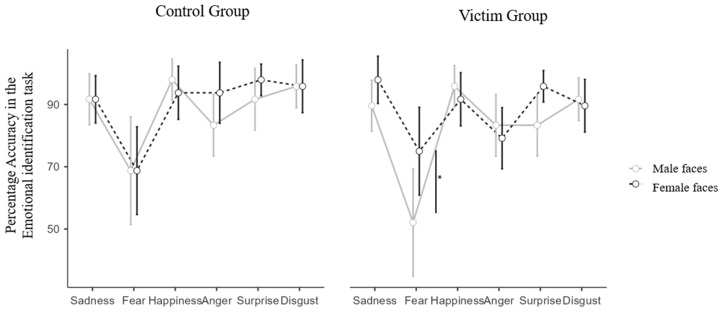
Mean values in the emotional identification task are represented for the male and female faces in the control and survivor groups. The error bars refer to the standard errors of the group mean, displayed with white circles. * *p* < 0.05. ** *p* < 0.005. *** *p* < 0.001.

Bonferroni’s corrected post hocs revealed that, only in the survivors group, the fearful male faces are worst recognized compared to the female ones (t (46) = 4.20, *p* = 0.010), while this contrast is non-significant within the control group, showing similar accuracy in identifying the male and female fearful faces. A visualization of the results can be found in Figure 4.

#### 3.2.2. TAS-20, PTSD Checklist, and BDI

We included in all our statistical models TAS-20, PTSD checklist, and BDI scores as covariates. However, the variance explained by the models where these factors were included as covariates was lower than in the models without these factors. For this reason, we conducted an exploratory follow-up analysis to explore possible differences in scores on these scales between the survivors and controls. We ran three different independent samples *t*-tests. We found, only in the PTSD scale, that the survivors (M = 35.71; SD = 18.72) had higher scores than controls (M = 23.25; SD = 16.87), t_(46)_ = −2.42, *p* = 0.02, and d = 0.699. No statistically significant differences were found in TAS-20 and BDI scores between the survivors and controls (all *p* > 0.10).

## 4. Discussion

The present study aimed to investigate trustworthiness and emotion perception in women who experienced IPV (IPV survivors or IPV-S) and the ones not having experienced violence in their lifetime (i.e., no-IPV) to explore the influence of IPV victimization on visual perception. The participants completed an online protocol where both tasks on trustworthiness and emotion perception were presented. The IPV-S were recruited through anti-violence centers, while the controls were volunteers who were screened as not having experienced IPV during their lives through an initial questionnaire. Subsequently, participants were asked to complete a preference judgment of perceived trustworthiness tasks, an explicit judgment of perceived trustworthiness, and emotional identification tasks. Potential symptoms of alexithymia, PTSD, and depression were measured by administering the corresponding questionnaire.

We found a series of novel results, starting from the analysis of the explicit judgment of the perceived trustworthiness tasks. Specifically, we found a correspondence between the perceived and presented levels of trustworthiness. Moreover, in general, the female faces were perceived as more trustworthy compared with the male faces. This result, however, occurred irrespective of the experimental groups. This is also in line with studies that have consistently shown that women are better at recognizing the faces of other women compared to men. The superiority in female-to-female facial recognition implies that women may possess a greater ability to discern facial characteristics among individual females [66,67]. Especially for trustworthiness from faces, recent studies have shown that female faces are perceived as more trustworthy than male faces [68,69,70]. Indeed, male faces are considered more threatening, displaying increased anger and greater potential for exploiting trust, activating a vigilance system.

In addition, the general tendency in women to perceive other women as more trustworthy might be greatly influenced by societal conditions. Wessells and Kostelny [71] argue that the psychosocial impact of IPV extends beyond direct survivors, affecting community norms and perceptions. This societal backdrop may contribute to women generally perceiving men as less trustworthy or more dangerous, even if they are not direct IPV-S. This context aligns with our findings and suggests that such perceptions are not merely a result of personal experience but are also shaped by broader societal conditions.

To further check whether this difference might be influenced by experiencing IPV, we conducted an exploratory analysis revealing that the IPVs rated highly trustworthy male faces as less trustworthy than the controls. This is in contrast with Perizzolo Pointet and colleagues [47] who found IPVs with PTSD to overestimate avatar faces on the trustworthiness dimension, judging faces as more trustworthy. Conversely, we found an underestimation of trustworthiness specific to the male faces in our experimental group. It should be noted that these inconsistencies between our results and those of Perizzolo Pointet and colleagues [47] may well be explained by the fact that the authors only used male faces, not allowing for a direct comparison between genders as we did. In addition, Perizzolo’s stimuli were computer-generated faces [72], and the participants may have overestimated their perceptual expertise at telling faces apart because they lack ecological validity. Indeed, the faces used in our study are also considered by adult evaluators to be more alike in appearance than those from the Todorov set, utilized by Perizzolo Pointet and colleagues [47]. This methodological aspect is particularly relevant, as the use of computer-generated faces may engage different cognitive and affective processing mechanisms compared to real faces. Indeed, previous research suggests that trustworthiness judgments tend to be lower for computer-generated faces compared to real faces, particularly for male faces rather than female faces, possibly due to their lack of fine-grained facial cues and lower ecological validity [73]. Consequently, the use of real faces in our study may have elicited more naturalistic trustworthiness judgments, making the results less susceptible to biases linked to artificial stimuli. Thus, the different results of these studies could be attributed to differences in the stimulus materials used and may need further studies.

Also, Perizzolo and colleagues explained these results as a consequence of the habituation process: women who have been directly exposed for a long time to partners and attachment figures with dominant personalities tend to underestimate the dominance and overestimate the trustworthiness in the faces. Perizzolo and colleagues also describe these results in terms of the possible activation of a protective mechanism to survive violence by behaving submissively [47]. It must be noted that this explanation should be interpreted cautiously. Indeed, a main factor impacting facial perception could also be the time from the violence to the test time. The authors of the study analyzed [47,74] recruited participants through flyers (self-reported IPV-s); thus, they did not exclusively recruit them from violence centers, as we did (vs other-reported IPV-s). Their sample is plausibly more heterogeneous from the perspective of trauma processing, abuse severity, and recurrence, and its impact on the women. On the contrary, in the present study, women undergo psychological and legal support to cope with or exit from the condition of violence. We acknowledge that the context of the IPV group—comprising women living in group facilities—may introduce specific factors that limit the generalizability of the findings to all IPV survivors. Thus, it might be that our sample is further from experiencing violence. As a consequence, they might judge the male faces as less trustworthy because they have learned not to trust them. Women who are no longer experiencing violence, possibly due to trauma, may develop diminished trust in abusive male partners, leading to a reduced perception of trustworthiness towards men in general. High levels of hypervigilance and PTSD symptoms are common among survivors of IPV [31], which can influence their perception of trust. Additionally, psychotherapy may help survivors of IPV recognize patterns of excessive trust in dysfunctional male figures from their past and reduced general trust in men going forward.

**Figure 5:**
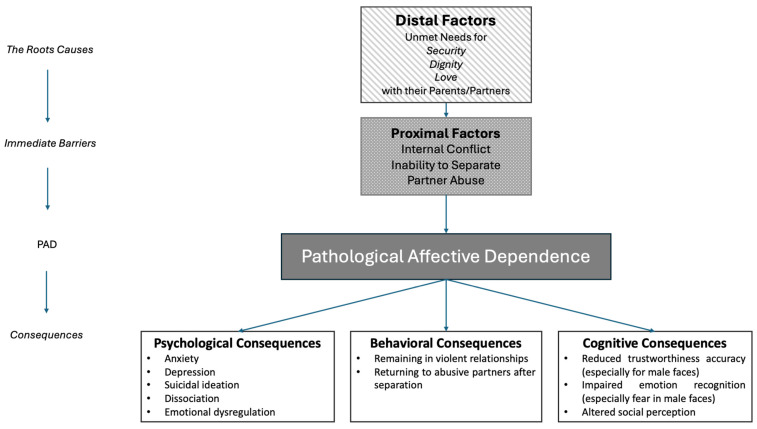
Visual representation of causal factors, PAD, and psychological, behavioral, and cognitive consequences.

In contrast, IPV-S who have not yet received help or treatment, or who are still in a situation of violence, may perceive the male faces as more trustworthy as a coping mechanism to maintain their abusive relationships. This aligns with PAD theory [20], which suggests that survivors of IPV may endure significant personal risk to preserve the relationship and avoid the distress of separation, as a consequence of violence in the relationship. From this perspective, PAD symptomatology may moderate the relationship between trust perception and PTSD symptoms. Specifically, individuals with high levels of PAD may be more likely to mistrust others while simultaneously experiencing an overwhelming need for connection and fear of abandonment. This internal conflict may intensify trauma-related symptoms, as the inability to reconcile distrust with the need for closeness could lead individuals to remain in a violent relationship or return to it after a separation. Figure 5 provides a visual representation of the relationship between the causal factors, PAD, and its psychological, behavioral, and cognitive consequences. This repeated exposure to harm may, in turn, exacerbate PTSD symptomatology, reinforcing a cycle of trauma and relational entrapment. Future studies could test this moderation effect explicitly, providing a more nuanced understanding of how relational dependency shapes trauma responses in the aftermath of IPV. Research should also explore how ongoing exposure to violence versus recovery from it affects the perception of the male faces as trustworthy. While evidence indicates that the negative effects of violence can have long-lasting impacts [31,75], it is plausible that perceptual mechanisms related to trustworthiness might recover sooner, potentially reaching levels comparable to those of individuals who have not experienced violence.

Thus, timing factors can contribute to face judgments, including the exposure for a long period of time to violence, the time that has passed since the last episode of experienced violence, and also the time between the violence and the psychological support. Thus, future studies should also take into consideration these factors, also assessing the participants’ history with IPV and other forms of violence to better understand the influence of such experiences on their social perceptions of facial cues.

This is consistent with studies highlighting higher cortisol levels in IPV-S compared to controls [76]. In IPV conditions, it could be that cortisol levels remain high to “help” survivors in responding to violence and facing it, potentially reducing the psychological and physical damage. Consistent with our speculation on the possible effect of time, it might be that those effects are visible in IPVs still in the abusive relationship, and, on the contrary, this might change when they are in a post-trauma phase, as is our case.

Our results, showing that IPV-s rate high-trustworthy male faces as less trustworthy than the controls, also align with previous studies on other types of violence [77,78,79], stating that traumatized patients and IPVs are “hypersensitized” to the identification of non-verbal bodily signals to recognize danger. Therefore, they tend to read every signal as more threatening. Similarly, traumatic experiences during childhood impacted trust perception with higher self-reported levels of distrust among individuals with childhood maltreatment across various age groups [80,81,82,83], as well as a lower ability to discern which individuals are trustworthy in trust games [84].

The results regarding the pairwise preference task highlight a significant main effect of the gender of the faces, with the participants (i.e., both the controls and IPVs) less accurate with the male compared to female faces. However, no main effect on the group was observed. A further explorative analysis aligns with our results on the explicit judgment task in revealing a tendency of IPVs to perform more poorly with male faces compared to controls.

When looking at the results regarding the emotional identification task, a higher accuracy with female than male faces was found, consistent with the previous results. However, a difference was observed in both groups. As in previous studies [67,85,86], the female participants are better at recognizing female faces. Although the mechanism behind this finding is still unclear, it is plausibly related to the extent or intricacy with which males and females process the faces of each gender, suggesting a more thorough or detailed processing of female faces (i.e., the previously discussed superiority in female-to-female facial recognition).

Moreover, fearful faces were recognized less accurately in the entire group, consistent with difficulty in recognizing fearful faces observed in the general population [56]. The evidence gathered from the present study adds to the existing literature, suggesting that fearful faces, compared to other emotions, are more difficult to recognize for both IPVs-S and controls, although gender differences may play a crucial role. An interesting result that emerged from our analyses is that IPVs were less accurate in identifying fear from male compared to female faces. This might be due to the fact that fear is already more difficult to recognize in general (as the main effect of emotion showed), and IPVs are less accurate in interpreting facial states in general, as in previous studies [46], and as our previous result on tendencies with trustworthiness judgments showed. Thus, it might be that the difficulty with fear recognition observed for all the participants was enhanced in the IPV group with the male faces. In this sense, this result is in line with the previously described result on a tendency of IPVs to be less accurate in identifying trustworthiness from male faces.

In conclusion, it is plausible that IPVs may have a specific deficit in recognizing trustworthiness from male faces, together with fear emotion. It seems that experiences with abusive individuals, specifically with abusive male figures, result in difficulties for the IPVs in recognizing whether the other person is trustworthy or their emotional state (particularly fear). This mechanism may be linked to the model of PAD [19,20], where the difficulty in breaking free from the abusive partner could be driven by the challenge of discerning whether the other person is worthy of trust or not. Indeed, as demonstrated and confirmed in recent studies by Pugliese et al. [19,20], a key aspect of this condition is the internal conflict experienced by IPVs. This conflict arises between the desire to save their own lives by separating from the abusive partner and the desire to preserve the relationship at all costs, despite the severe consequences of remaining in the relationship. This internal mental struggle is a result of the abuse and is exacerbated by the survivors’ perceived inability to leave the abusive partner and the ongoing perception of the partner’s abuse. The abuse itself intensifies this conflict, particularly because the abuser, who is objectively threatening, is also the person who should be providing protection to the survivor. The repeated exposure to that kind of relationship pattern pushes the IPVs to question the true identity of the abuser, which translates into the difficulty in recognizing what is trustworthy and what is not, and what is safe and what is threatening.

Future studies could shift their focus to not only trustworthiness perception from faces but rather into actual trust behaviors, assessed through trust games [87,88]. Indeed, being able to overcome face-based trust initial impressions has a highly socially adaptive value in potentially dangerous situations in which someone trustworthy-looking behaves as untrustworthy. Lastly, the results regarding alexithymia and depressive symptoms showed no significant variations between the two groups concerning these variables. However, the IPVs exhibited significantly higher levels of PTSD symptoms compared to the controls. In line with the existing literature [89], IPVs usually manifest PTSD symptoms, specifically following psychological abuse. However, the low variance explained when including these factors as covariates underscores the importance of directly examining the group differences in relevant psychological measures, providing valuable insights into the specific impact of traumatic experiences on mental health outcomes. This suggests that future research could benefit from better exploring the traumatic significance of the violent experience, and expanding the sample size, which is relatively low in the current sample due to recruitment reasons. Indeed, the small sample size represents a limitation for drawing a clear interpretation of our results. While our findings provide initial evidence on altered trustworthiness perception in IPV survivors, the limited sample size affects the robustness and generalizability of these results. Larger-scale studies are crucial to confirm and expand upon these findings, allowing for a more comprehensive understanding of how trauma influences face perception and trust-related behaviors. Additionally, future research should consider the potential impact of participants’ variability in age and lived experiences, which may introduce additional factors influencing these cognitive and perceptual processes. More studies with a greater sample size are needed to confirm and extend these results. Also, the literature on trustworthiness perception employs a wide variety of stimuli, ranging from validated naturalistic faces to computer-generated faces. This heterogeneity in stimulus selection impacts the consistency and comparability of findings across studies, as different types of stimuli may engage distinct cognitive and perceptual mechanisms. Although the use of KDEF faces is well-established and their trustworthiness levels are derived from normative validations (e.g., Chicago Face Database), we acknowledge that these validations were not conducted specifically within an IPV population. Our study provides the first evidence of altered trust perception among survivors of IPV. Future research with larger sample sizes could further strengthen and expand upon these findings, also investigating whether other facial cues (e.g., attractiveness, dominance, threat, etc.) embedded in these stimuli are similarly perceived in populations with traumatic experiences.

Our result encourages further investigation into the relationship between face perception and traumatic and violent experiences, specifically the relation between trustworthiness perception and the actual behavior of trust in IPVs. The aim is to better frame these considerations within the PAD theory by Pugliese et al. [19], when IPV-S, like in a game of tug-of-war, are often pulled in different directions by their experiences by an internal conflict between the goal to separate from their abusive partner and the goal to remain or return to the abusive partner.

These results should also be taken into account by the government and non-government organizations to adequately respond to the deep-rooted and highly complicated gender inequality by implementing preventive measures and providing comprehensive rehabilitative services for the IPV-S. Additionally, future researchers should adopt longitudinal designs to establish causal relationships and qualitative studies to provide a more in-depth understanding of the context and drivers of IPV.

## Data Availability

The data that support the findings of this study are available in the OSF at the following link: https://osf.io/t69he/?view_only=196e15e2fdd647b681a64233d03ebe0c accessed on 29 August 2024.
